# The Effect of Diet on Vascular Aging: A Narrative Review of the Available Literature

**DOI:** 10.3390/life14020267

**Published:** 2024-02-17

**Authors:** Xenophon Theodoridis, Michail Chourdakis, Androniki Papaemmanouil, Stavroula Chaloulakou, Athina Vasiliki Georgakou, Georgios Chatzis, Areti Triantafyllou

**Affiliations:** 1 Laboratory of Hygiene, Social and Preventive Medicine and Medical Statistics, School of Medicine, Faculty of Health Sciences, Aristotle University of Thessaloniki, 54124 Thessaloniki, Greece; xtheodoridis@auth.gr (X.T.); acpapaem@auth.gr (A.P.); chstav@auth.gr (S.C.); georathi@auth.gr (A.V.G.); 2 Third Department of Internal Medicine, Papageorgiou Hospital, Aristotle University of Thessaloniki, 56403 Thessaloniki, Greece; 3 School of Physical Education and Sports Science, Aristotle University of Thessaloniki, 57001 Thessaloniki, Greece; dietchatzis@gmail.com

**Keywords:** vascular health, dietary regimens, arterial stiffness, endothelial function, Mediterranean diet, caloric restriction, DASH diet

## Abstract

Early vascular aging is related to various cardiovascular diseases including hypertension, coronary heart disease, and stroke. Healthful lifestyle practices and interventions, including dietary regimens and consistent aerobic exercise, exert favorable modulation on these processes, thereby diminishing the risk of cardiovascular disease with advancing age. The principal objective of this review was to conduct a comprehensive evaluation and synthesis of the available literature regarding the effectiveness of different diets on vascular health, such as arterial stiffness and endothelial function. To conduct this review, a thorough search of electronic databases including PubMed, Scopus, and Web of Science Core Collection was carried out. Based on the existing evidence, the Mediterranean, Dietary Approaches to Stop Hypertension, and low-calorie diets may have a beneficial effect on vascular health. However, more randomized controlled trials with sufficient sample sizes, longer follow-ups, rigorous methodologies, and, possibly, head-to-head comparisons between the different diets are needed to shed light on this topic.

## 1. Introduction

The pathogenesis of numerous chronic diseases is posited to be heavily influenced by aging, an intricate multifactorial process characterized by gradual dysregulation and diminution of function across diverse levels and systems [1]. By the year 2030, it is projected that one in six individuals globally will have reached the age of 60 or older. Furthermore, by 2050, the worldwide population of individuals aged 60 and older is expected to double, reaching 2.1 billion. Notably, the demographic cohort of individuals aged 80 or older is foreseen to undergo a threefold increase between 2020 and 2050, reaching a total of 426 million [2].

Thomas Sydenham reported that “a man is as old as his arteries”, implying that vascular age may be more crucial than chronological age in identifying the risk of developing diseases [3]. The predominant feature of vascular aging is the alteration in the mechanical and structural properties of the vascular wall [4]. Early vascular aging (EVA), a term coined by Nilsson and their colleagues [5], is associated with the premature onset of a plethora of cardiovascular diseases (CVD), including hypertension (HTN), coronary heart disease (CHD), and stroke [6]. Quantifying EVA has been demonstrated as a valuable strategy and holds potential significance in guiding forthcoming preventive and therapeutic interventions [7].

Significantly, healthy lifestyle practices and interventions, including various dietary regimens and consistent aerobic exercise, positively influence the above-mentioned processes, thereby diminishing the risk of CVD with advancing age [8]. For example, published studies indicate that the Mediterranean diet (MD) and the Dietary Approaches to Stop Hypertension (DASH) diet may have a favorable effect on arterial stiffness (AS) [9,10]. Moreover, caloric restriction (CR) is among the most influential strategies for extending maximal lifespan and health span in rodents [8].

To the best of our knowledge, to date, there is no comprehensive review consolidating published articles pertaining to the role of the majority of studied dietary patterns on AS and endothelial function (EF). Hence, the aim of this narrative review was to summarize the existing evidence regarding the effect of different dietary patterns on vascular health.

## 2. Materials and Methods

The main objective of this review was to perform a comprehensive evaluation and synthesis of the available body of research regarding the effectiveness of different diets on vascular health, such as AS and EF. The review endeavors to furnish an up-to-date and thorough understanding of the dietary management of vascular outcomes including, among others, pulse wave velocity (PWV), augmentation index (AIx), and flow-mediated dilatation (FMD).

A search of electronic databases including PubMed, Scopus, and Web of Science Core Collection was carried out to cover the medical literature comprehensively. The search process involved utilizing combinations of Medical Subject Headings (MeSH) terms and keywords including MD, DASH diet, vegetarian diet, Western diet, and CR with the integration of Boolean operators to refine the search queries. The detailed search strategy for the PubMed database can be found in the Supplementary Materials (Supplementary Table S1).

The inclusion criteria for the present narrative review were peer-reviewed articles, systematic reviews, randomized controlled trials (RCTs), clinical trials (CT), and observational studies assessing the effects of different dietary patterns including the MD, DASH diet, vegetarian diet, low-fat diet, low-carbohydrate diet, and Western diet on vascular health and EF.

We excluded studies with pregnant populations, those written in languages other than English, and those published before 2000.

## 3. Dietary Patterns

### 3.1. Mediterranean Diet

The MD consists of a traditional dietary pattern of the Mediterranean region that has been characterized by the high consumption of seasonal fruits and vegetables, nuts, cereals (mostly whole grain), legumes, olive oil, and fish, moderate consumption of alcohol and dairy products, and low consumption of meat and meat products [11,12]. In a broader sense, the Mediterranean way of life includes resting patterns [13] and psychosocial factors, like fostering friendships and savoring the company of friends, and also highlights the importance of physical activity [14].

Several studies have demonstrated the MD’s crucial role in risk reduction of cardiovascular and metabolic diseases [15] and healthy aging promotion [16]. Research on the elderly has revealed that following the MD may enhance endothelial-dependent vasodilatation by increasing NO bioavailability [17]. The MD also seems to be related to the reduction of inflammation, as well as the improvement of lipid profile, insulin sensitivity, and glucose metabolism, which contribute to the delay of the aging process [16]. Additionally, the MD seems to prevent the so-called “inflammaging” by maintaining gut microbiota homeostasis or by acting through the epigenetic mechanism, which involves chromatin remodeling, DNA methylation, and microRNAs (miRNAs) [13]. Regarding endothelium health, the MD has been recognized as an important factor in the endothelium’s recovery and regenerative capacity, as well as a protector of endothelium dysfunction due to its greater antioxidant capacity [18]. Additionally, brisk walking exercise, which is a component of the wider Mediterranean way of life, has been associated with improved microvascular EF [19]. The key compounds and sources associated with the antioxidative effects of the MD include polyphenols, extra-virgin olive oil, antioxidant vitamins such as vitamins A, C, and E, minerals including magnesium, potassium, and selenium, flavonoids, phytochemicals, and fiber [20].

Lobene et al. [21] conducted a cross-sectional study in healthy young adults with no history of CVD, examining the correlation between the alternative Mediterranean diet score (aMED) and AS, evaluated by PWV and AIx. In their study, EF was also assessed using the FMD. Their results showed an inverse association between aMED and AIx (β = −1.59, 95% CI: −3.09 to −0.09). PWV and FMD were not correlated with the aMED (β = 0.02, 95% CI: −0.09 to 0.13; β = −0.24; 95% CI: −0.60 to 0.12, respectively). When the sex interaction term was added to the regression model, no associations were found between aMED and the aforementioned indexes (AIx: β = 1.40, *p* = 0.40; PWV: β = 0.06, *p* = 0.62; FMD: β = 0.17, *p* = 0.66).

EVIDENT was a cross-sectional multicenter study in which PWV, carotid intima-media thickness (cIMT), and biological markers of endothelial dysfunction were evaluated as primary measurements [22]. Adherence to the MD was assessed using the 14-point Mediterranean diet adherence screener (MEDAS) questionnaire. The investigators showed a negative association between the healthy diet score and the PWV (β = −0.145, *p* = 0.034), as well as the radial AIx (β = −0.262, *p* = 0.035). No associations were observed between the healthy diet score and the central AIx (β = −0.202, *p* = 0.202), nor the ambulatory arterial stiffness index (β = −0.032, *p* = 0.833) [23]. In another study from the same protocol, the EVIDENT diet index, which was developed by the investigator group, was found to be a predictor of MD adherence. According to the multiple regression analysis, every 1-point increase in the EVIDENT index was associated with a decrease in PWV (β = −0.082, *p* = 0.014; β = −0.089, *p* = 0.003 for the two adjusted models) [24].

In the study by Rallidis et al. [25], patients with abdominal obesity (AO), free from CVD or type II diabetes mellitus (T2DM), were randomized to receive a simple consultation (control group) or a more extensive training on the MD (intervention group). FMD was the assessed marker of EF and the findings showed that FMD was increased in the intervention group compared with the control group (*p* = 0.042).

The CORDIOPREV study was a prospective randomized single-blind controlled trial including patients with CHD, in which the impact of a low-fat versus a Mediterranean diet was compared. In this study, EF was assessed using the FMD. Even in patients with severe endothelial dysfunction, participants following the MD had higher FMD (3.83%, 95% CI: 2.91 to 4.23) than the patients on the low-fat diet (1.16%, 95% CI: 0.80 to 1.98) with a difference of 2.63% between the aforementioned dietary patterns (95% CI: 1.89 to 3.40) [26]. Another publication related to the CORDIOPREV study indicated that the MD enhanced FMD in patients with diabetes (5.2 ± 0.4% at 1.5 years follow-up vs. 3.8 ± 0.4% at baseline; *p* = 0.04) and prediabetes (4.9 ± 0.4% at 1.5 years follow-up vs. 3.8 ± 0.4% at baseline; *p* = 0.04). Additionally, the MD was associated with improved endothelial activity after a 1.5-year time interval in patients with diabetes (5.2 ± 0.4 vs. 3.7 ± 0.4; *p* = 0.01) compared with the low-fat diet [27].

The study by Gómez-Sánchez et al. [28] was a cross-sectional, descriptive, multicenter study, part of the MARK Study, including people with moderate cardiovascular risk. Subjects enrolled in this study had their dietary intake evaluated by the diet quality index (DQI) questionnaire and the MD adherence questionnaire. According to the findings from the multiple regression analysis, for each 1-point increase in the DQI score, a non-significant decrease of −0.081 (95% CI: −0.105 to 0.028) in brachial–ankle PWV (baPWV) was observed. Accordingly, as the MD adherence questionnaire score increased by 1 point, there was a decrease in baPWV (β = −0.052, 95% CI: −0.141 to −0.008). In the sex-based analysis, the association held up only in men for both DQI and MD questionnaires (β = −0.118, 95% CI: −0.145 to −0.054 and β = −0.081; 95% CI: −0.198 to −0.04, respectively). Furthermore, in the same study, it was shown that the greater the MD adherence, the lower the odds of presenting EVA, both in the DQI (OR = 0.65, 95% CI: 0.56 to 0.97) and MD adherence questionnaire (OR = 0.75, 95% CI: 0.58 to 0.97). In the sex-based analysis, the association held up only in men (DQI: OR = 0.54, 95% CI: 0.39 to 0.76; MD: OR = 0.71, 95% CI: 0.52 to 0.99) [28].

A previous study from the same group of investigators, titled the EVA study, included individuals free from CVD and was designed as a descriptive transversal study. Adherence to the MD was assessed using the 14-item MEDAS questionnaire. The study population was categorized based on age and sex using the vascular aging index (VAI) percentiles. The VAI was calculated using the cIMT and the carotid–femoral PWV (cfPWV). The results indicated that participants with EVA presented a lower percentage of MD adherence (2%) than those with normal vascular aging (NVA). According to the adjusted logistic regression analysis, as MD adherence increases, the odds of EVA decrease (OR = 0.36, 95% CI: 0.16 to 0.82) [29].

The MEDITA trial, published by Maiorino et al., consisted of a parallel two-arm single-center RCT, enrolling patients with newly diagnosed T2DM. Participants, who were free from apparent CVD, were randomized to receive either an MD, containing less than 50% of calories from carbohydrates and more than 30% of calories from fat, or a low-fat diet, including less than 30% of calories from fat. At the 4-year evaluation, as well as at the evaluation conducted at the end of the trial, a difference favoring the MD (MD = −0.019, 95% CI: −0.035 to −0.003; MD = −0.025, 95% CI: −0.040 to −0.005; respectively) was observed [30].

Jennings et al. [9] aimed to determine the effect of an MD-style diet on AS, assessed by AIx and PWV in participants of the NU-AGE study [31]. This study was a single-blind RCT, with two parallel arms (control vs. “diet group”), that lasted 12 months and took place in five European centers, and included older adults. Participants in the intervention group were provided with personalized dietary suggestions and commercially available food in order to increase their compliance with the MD. The intervention did not result in improved AIx (MD = −6.1, 95% CI: −12.5 to 0.3). However, a between-group difference of −12.4 (95% CI: −24.4 to −0.5) was observed, indicating an improvement in AIx resulting from the intervention. No differences were found regarding PWV (*p* = 0.6) [9].

In the study performed by Klonizakis et al. [19], 22 healthy participants were randomized to either receive an MD or non-MD dietary pattern followed by an 8-week exercise intervention. Their findings showed no difference between the two groups in endothelium-dependent vasodilation following the 8-week period (*p* = 0.25).

The RoCAV study was a cross-sectional cohort study with participants randomly selected from Northern Italy without main chronic diseases [32]. For study purposes, the investigators performed a principal components analysis (PCA) in order to identify dietary patterns in the study population. PC1 was found to be equivalent to a Western-type diet, whereas PC2 was characterized as being like the MD. The adherence to the MD was also calculated using the Mediterranean diet score (MedS). CfPWV was evaluated as a marker of AS. In the model adjusted for age, sex, and energy intake, PC2 did not present an association with cfPWV (β = −0.18, 95% CI: −0.36 to 0.01). Additionally, in the second model, which comprised Model 1 further adjusted for cigarette smoking and educational level, as well as in the third model, which was Model 2 further adjusted for body mass index (BMI), HTN, and dyslipidemia, no associations were observed (β = −0.18, 95% CI: −0.36 to 0.004; β = −0.12, 95% CI: −0.30 to 0.05). The same findings applied to the fourth model, which was Model 2, further adjusted for glucose and leucocytes (β = −0.17, 95% CI: −0.35 to 0.01). Furthermore, none of the levels of MD adherence presented an association with cfPWV, in any model [33].

Angelis et al. [34] conducted an epidemiological study in male patients with chronic heart failure in which compliance with the MD was evaluated using the 11-item MedDietScore. Data analysis revealed a negative association of the MedDietScore with AIx (β = −0.116, *p* = 0.014), whereas no association was observed between the aforementioned index and the PWV (β = −0.073, *p* = 0.37).

Lee and colleagues [35], in a controlled crossover study, evaluated the association of a 10-day MD versus a typical diet on the AIx in a sample of healthy women. Investigators showed an absence of association between the diet intervention and the aforementioned index (MD = 2.50, *p* = 0.13), whereas an association was found between the diet intervention and the augmentation pressure (MD = 6.15, *p* = 0.02).

Regarding the MedLey RCT with two parallel groups [36], healthy older adults were allocated to receive either an MD or a habitual diet for a period of 6 months. BaFMD was measured as a marker of EF. The mean FMD at 6 months was higher in the MD group than in the habitual diet group (2.5% vs. 1.2%, *p* = 0.03).

The study by Murie-Fernandez and colleagues [37] was part of the PREDIMED RCT and consisted of high-cardiovascular-risk asymptomatic participants who were assigned to three arms: MD supplemented with extra-virgin olive oil (MD+EVOO), MD supplemented with nuts, and a control diet. The primary endpoint was the 1-year between-group change in cIMT. The 1-year cIMT presented a difference only in the MD+nuts group (mean = −0.031, 95% CI: −0.055 to −0.007). When the sample was divided according to baseline cIMT (cut-off: 0.9 mm), reductions were shown for the MD+EVOO and MD+nuts groups, only among participants with baseline cIMT ≥ 0.9 mm (mean = −0.093, 95% CI: −0.146 to −0.039; mean = −0.086, 95% CI: −0.138 to −0.034) [37].

Shannon et al. [38] conducted a systematic review and a meta-analysis including 14 RCTs related to the impact of the MD on EF and FMD, including a total of 1930 subjects. The findings showed an improvement in EF (SMD = 0.35, 95% CI: 0.17 to 0.53). More specifically, in the subgroup analyses, the MD was found to be related to EF improvement in healthy subjects (SMD = 0.29, 95% CI: 0.05 to 0.53), as well as in people with increased risk of CVD (SMD = 0.36, 95% CI: 0.15 to 0.58). It was also observed that the MD increased FMD by 1.66% (95% CI: 1.15 to 2.17).

Associations between the MD and vascular health have also been investigated in children and adolescents, as presented in the study by Lydakis and colleagues [39]. In their observational study, AIx was measured as a marker of vascular health and adherence to the MD was assessed using the Mediterranean Diet Quality Index for Children and Adolescents (KIDMED) score. According to the multiple regression analysis results, the KIDMED index showed an inverse association with AIx (β = −0.114, *p* = 0.026) [39].

The characteristics of the included studies assessing the effect of the MD on vascular health markers are presented in Table 1.

### 3.2. DASH Diet

The DASH diet is a dietary pattern involving a high consumption of fruits, vegetables, whole grains, fish, nuts, dairy products, and vegetable oils, as well as a low consumption of processed meat, sugary products, and alcohol [40]. Among its special characteristics, it is established that its content of saturated fat and cholesterol is low, in conjunction with low sodium intake (<2300 mg/d), making it a beneficial dietary pattern for ameliorating CVD outcomes [41]. The key distinctions between the DASH and Mediterranean diets lie in their focal points. The DASH diet places a primary emphasis on reducing sodium intake, while the MD centers around overall dietary patterns [42]. The MD promotes the consumption of extra-virgin olive oil, in contrast to the DASH diet, which underscores the consumption of low-fat products. Furthermore, the MD encourages moderate alcohol consumption, while the DASH diet recommends the elimination of alcohol intake [42]. Additionally, the DASH diet provides more specific recommendations for certain nutrients such as potassium, sodium, and calcium, whereas the MD adopts a broader approach, emphasizing a variety of nutrient-dense foods [42].

Due to the described composition of the DASH diet, several beneficial effects on health outcomes and biomarkers have been identified, such as improving blood pressure and lipids, while in parallel reducing inflammation [40] and oxidative stress. These mechanisms seem to be associated with reduced AS and improved endothelium-dependent dilation [43].

The primary outcomes of the DASH trial pertain to the alterations in systolic and diastolic blood pressure among adults with systolic blood pressure of less than 160 mm Hg and diastolic blood pressure ranging from 80 to 95 mm Hg [44]. The trial evaluated the impact of three dietary interventions: a control diet low in fruits, vegetables, and dairy products, a diet rich in fruits and vegetables, and a “combination” diet enriched with fruits, vegetables, low-fat dairy products, and reduced saturated and total fat content. The findings showed a reduction in both systolic and diastolic blood pressure, indicating that the “combination” diet was effective in lowering blood pressure levels for participants with both hypertension and normal blood pressure compared to the other two groups [44].

The ENCORE study was designed as an RCT, including overweight and above-normal BP subjects. Participants were randomized to the “DASH diet alone” intervention (DASH-A), the “DASH diet combined with a behavioral weight management program” (DASH-WM), or the usual diet as a control group. Blumenthal et al. showed that the implementation of the DASH diet—alone or accompanied by caloric reduction—was not associated with larger improvements in FMD (*p* = 0.06), but with lower PWV (*p* = 0.001) compared to the control group. PWV was lower in the DASH-WM group when compared to the DASH-A group (*p* = 0.045) [45].

In the study by Lobene et al. [21], which was mentioned before, dietary quality was assessed using the Mellen DASH score and the Fung DASH score. The aforementioned scores were not associated with any of the AIx (β = −0.99, 95% CI: −3.25 to 1.26; β = −0.36, 95% CI: −1.02 to 0.31, respectively), PWV (β = −0.003, 95% CI: −0.16 to 0.15; β = −0.006, 95% CI: −0.05 to 0.04, respectively), and FMD (β = −0.33, 95% CI: −0.85 to 0.19; β = −0.11, 95% CI: −0.25 to 0.04).

Gauci et al. [46] performed an exploratory cross-sectional study, including participants in the Memory and Attention Supplement Trial (MAST) RCT. They examined participants’ adherence to the MD, DASH diet, and Mediterranean-DASH Intervention for Neurodegenerative Delay (MIND) diet using a 14-item MD assessment tool, an 11-item score specific to the DASH diet, and a 15-item score particular to the MIND diet, respectively. PWV and AIx were also assessed as markers of vascular health. According to the results, only adherence to the DASH diet was related to lower AIx (β = −0.17, *p* = 0.032). However, it is important to note that this relationship did not remain after the sensitivity analysis (β = −0.15, *p* = 0.085) [46].

Maddock and colleagues [47] investigated the relationship between DASH diet adherence and cfPWV and cIMT in a sample of participants from the Medical Research Council (MRC) National Survey of Health and Development (NSHD). The Fung index was utilized in order to compute the DASH-type diet score. The findings indicated that there was an association between the standardized cIMT (β = −0.35, 95% CI: −0.54 to −0.16) and the Q5, which was the quartile with the highest adherence to the DASH diet. Accordingly, an association was found between the Q5 and the standardized PWV (β = −0.3, 95% CI: −0.51 to −0.10). The results were related to the Model 1 of the analysis, which was adjusted for the socioeconomic position. The same pattern was also observed in the Model 2 (additionally adjusted for BMI, smoking, and physical activity) (cIMT: β = −0.24, 95% CI: −0.44 to −0.04; PWV: β = −0.28, 95% CI: −0.50 to −0.07). The difference was also preserved in the analysis adjusted for common cardiovascular risk factors (cIMT: β = −0.24, 95% CI: −0.44 to −0.04; PWV: β = −0.24, 95% CI: −0.45 to −0.04) [47].

The TRIUMPH RCT included a 4-month intervention where patients with resistant HTN were randomized with 2:1 allocation to either a Center-based Lifestyle Intervention (C-LIFE) or Standardized Education and Physician Advice (SEPA) [48]. Both interventions included instructions related to DASH diet implementation. Among secondary endpoints, baFMD and PWV were evaluated. Subjects in C-LIFE demonstrated improvements in FMD [0.3% (−0.3, 1.0)] compared to SEPA participants [−1.4% (−2.5, −0.3), *p* = 0.022]. However, no difference was observed regarding PWV [0.16 (−0.40, 0.72) vs. 0.14 (−0.72, 1.0) (*p* = 0.958)] [48].

In the RCT by Lin et al. [49], participants with stage 1 HTN were randomized to either receive a DASH diet or a control diet after 1 week of a run-in period in which they followed a typical American diet. Vascular EF and AS were assessed by baFMD, AIx, and PWV. There were no changes in baFMD or AIx throughout the study (*p* = 0.741, *p* = 0.270, respectively) for both the control and DASH diet groups. Nevertheless, there was a decrease in PWV over time in the DASH group (*p* = 0.019), whereas no change was observed for the control group (*p* = 0.437) [49].

The characteristics of the included studies assessing the effect of the DASH diet on vascular health markers are presented in Table 2. 

### 3.3. Vegetarian Diet

Vegetarian diets have been characterized by the reduction or elimination of animal products consumption. They are frequently high in grains, legumes, fruits, vegetables, and nuts and low in added sugars, salt, cholesterol, and saturated fat. Vegetarian diets are known for their important content of various antioxidants and phytochemicals, associated with reduced oxidative stress and inflammation, which are key contributors to vascular health. Vegetarian diets have also been linked to decreased blood pressure and lower concentrations of blood lipids [50].

Mayra et al. [51] conducted a cross-sectional study investigating the impact of a vegetarian diet on AS evaluated by the cfPWV. Healthy, non-smoking adult participants followed a vegan/vegetarian or an omnivore diet (a dietary pattern with a combination of plant-based and animal products). According to the results, cfPWV did not differ between the two groups (7.0 ± 1.5 in omnivores and 6.8 ± 1.1 m/s in vegan/vegetarians, *p* = 0.073).

In the cross-sectional study by Gonzalez and colleagues [50], vascular function assessed by baFMD and AS evaluated by cfPWV and AIx were compared between healthy vegetarians and omnivores. The findings of this study showed no difference between the two groups regarding the baFMD (*p* = 0.290), the PWV (*p* = 0.171), and the Aix (*p* = 0.569).

Chen et al. [52] conducted a prospective study measuring the cIMT in a sample of older adults, of which 52% were vegetarians. cIMT was found to be lower in the group of vegetarians (0.66 ± 0.19) compared to the rest of the participants (0.69 ± 0.19) (*p* = 0.004).

A single-center, cross-sectional study with healthy men was designed by Page and colleagues [53] in order to evaluate the difference between a vegan and an omnivorous dietary pattern on baFMD and cIMT, among other markers. According to the results of the study, neither baFMD nor cIMT differed between the two dietary patterns (95% CI: −2.84 to 5.27, 95% CI: −0.07 to 0.03, respectively) [53].

The characteristics of the included studies assessing the effect of the vegetarian diet on vascular health markers are presented in Table 3. 

### 3.4. Caloric Restriction

Caloric restriction refers to a dietary pattern in which an individual’s daily energy intake is reduced in relation to their normal consumption while ensuring adequate nutrition without the presence of malnutrition [54].

Intermittent fasting (IF) is an eating pattern characterized by alternating periods of feeding and fasting [55]. The most prevalent IF regimens include the 16/8 method, which entails daily fasting for 16 h and restricting the eating period to an 8 h window. Another approach is the 5:2 diet, where individuals eat normally for five days a week, while on the remaining two non-consecutive days, they restrict their calorie intake to around 500–600 calories. Lastly, alternate day fasting is a pattern that alternates between days of normal feeding and days of either partial or complete fasting [55].

Key mechanisms related to the association of CR and vascular health include the reduction of oxidative stress, modulation of inflammation, and amelioration of EF. The favorable outcomes of CR on vascular activity most likely derive from the stimulation of multiple energy-sensing cellular signaling networks, including sirtuin−1 (SIRT−1) and AMP-activated protein kinase (AMPK), and inhibition of pro-growth mediators such as the mammalian target of rapamycin (mTOR) [55]. On the other hand, the relationship between excess adipose tissue and endothelial dysfunction may be related to resistance to the vasomotor function of insulin and leptin, activation of the renin–angiotensin–aldosterone system (RAAS), and direct adverse effects of several adipokines and other vasoactive factors [56]. Intermittent energy restriction has additionally been linked to lipidemic profile improvement and blood pressure reduction, which may contribute to vascular health [57].

Alinezhad-Namaghi conducted a cohort study in adults with metabolic syndrome (MetS) in order to investigate the influence of Ramadan fasting (RF) on vascular indexes. They demonstrated a reduction in PWV (−0.29 ± 1.02 m/s; *p* = 0.014), arterial age (−6.80 ± 17.46; *p* = 0.001), % central Alx (−2.47 ± 10.19; *p* = 0.036), and central augmentation pressure (−1.88 ± 5.40; *p* = 0.003) after the intermittent fasting period in the RF group. In the Ramadan non-fasting group, no differences were found regarding PWV, arterial age, central AIx, and central augmentation pressure (*p* = 0.50, *p* = 0.40, *p* = 0.37, and *p* = 0.35, respectively) [58].

Headland et al. [59] published a short report of a 4-week randomized, single-blind, crossover design including 35 participants. People enrolled in the study consumed an extremely low-energy diet (500 calories for women and 600 calories for men) for two consecutive days, along with five days of regular eating (5:2 intermittent energy restriction diet plan). In weeks 3 and 4 when FMD was measured, blood samples were collected after two days of usual feeding or two days of CR in a randomized sequence. The results underscore an absence of difference in FMD when the two dietary patterns were compared (*p* = 0.7) [59].

Jefferson et al. [60] conducted an RCT with a 5 months duration, in which the effects of resistance training (RT) along with or without CR were compared. For the assessment of AS, they measured baPWV, as well as large and small artery elasticity. Investigators showed that baseline baPWV was higher in the group following the RT plus CR intervention (*p* = 0.01). The main analysis highlighted an absence of within-group changes in baPWV, large or small artery elasticity, systemic vascular resistance, or ankle-brachial index in either group [60].

Raitakari and colleagues [61] performed a trial including men and women with overweight or obesity following a 6-week weight reduction program induced by a very low-calorie diet (daily energy: 580 kcal). Their protocol included the measurement of FMD as a marker of EF. According to the findings, post-intervention FMD was found elevated by 60% (*p* < 0.001). It is important to mention that improvement in FMD moderately correlated with the decrease in plasma glucose concentration (r = 0.44, *p* = 0.0003) but not with changes in weight (r = 0.01, *p* = 0.92) or other risk factors [61].

According to the RCT by Gonçalinho et al. [62], endothelium-dependent FMD and endothelium-independent vasodilation (NMD) were assessed in a group of healthy subjects randomized to either receive a resveratrol supplement (500 mg/day) or a low-calorie diet (1000 kcal/day, providing over 50% energy restriction). An absence of difference was observed both for the FMD and the NMD (*p* = 0.443; *p* = 0.196, respectively), between the baseline and the post-treatment assessment, regarding the energy restriction group [62].

In the SR and MA by Petersen et al. [63], 20 studies were included with a total number of 1259 participants. Individual studies involved energy-restricted diets with or without physical activity sessions as part of the different interventions and evaluated aortic, brachial–ankle, carotid–femoral, or femoral–ankle PWV (faPWV). The overall result of the meta-analysis indicated that weight loss, either achieved by energy restriction or diet plus exercise, was associated with a reduction of PWV (SMD = −0.32, 95% CI: −0.41 to −0.24). Additionally, no difference was found between the two interventions (*p* = 0.66), and no difference was observed in the response to weight loss when the different types of PWV were compared (*p* = 0.11). Moreover, cfPWV (SMD = −0.35, 95% CI: −0.44 to −0.26) and baPWV (SMD = −0.48, 95% CI: −0.78 to −0.18) were found to be decreased by weight loss [63].

In the trial by Klempel and colleagues [64], the alternate day fasting (ADF) approach was studied in relation to baFMD. ADF is divided into two phases: the “feed day”, a 24 h time of unlimited food consumption, and the “fast day”, a 24 h period of 50–100% CR. The total duration of the trial was 10 weeks, which was separated into a 2-week baseline weight maintenance period and an 8-week weight loss period. In the intervention period, the two groups were randomly assigned to receive a high-fat- (ADF-HF) or a low-fat (ADF-LF) diet. According to the final results, a 1.8% reduction of FMD was observed (*p* < 0.05) in the ADF-HF group relative to baseline, whereas a 2.1% increase of FMD was detected (*p* < 0.05) in the ADF-LF group [65].

Weiss et al. [66] conducted a randomized intervention trial in which men and women with overweight were randomly assigned to achieve a weight reduction by 6−8% using a CR program, an endurance exercise program (EX), or a combination of both strategies (CREX). For the evaluation of the AS, PWV and AIx were measured. The investigators did not observe differences among PWV, neither within groups (All: *p* = 0.30; CR: *p* = 0.92; EX: *p* = 0.17; CREX: *p* = 0.39) nor between groups (*p* = 0.66). The same applied to the AIx in the within-group analysis (All: *p* = 0.84; CR: *p* = 0.68; EX: *p* = 0.86; CREX: *p* = 0.36), as well as in the between-group analysis (*p* = 0.6) [66].

Nordstrand and colleagues [67] conducted a non-randomized controlled trial (nRCT) in people with severe obesity in order to compare the 7-week effect of a low-calorie diet (LCD) and an intensive lifestyle intervention program (ILI), including an educational session on nutrition and physical activity, as well as training sessions, on AS. A decrease in mean PWV was observed in the ILI group (within-group difference = −0.6, 95% CI: −0.8 to −0.4), whereas in the LCD group, no difference was demonstrated (95% CI: −0.4 to 0.0). After the adjustment for age, gender, baseline mean arterial pressure, baseline BMI, history of coronary artery disease, and baseline PWV, the decrease in PWV was higher in the ILI group than in the LCD group, presenting a between-group difference of 0.4 m/s (95% CI: 0.1 to 0.6) [67].

The randomized, parallel-designed study by Figueroa et al. [68] consisted of post-menopausal (PM) women with overweight or obesity. They were randomly assigned to receive a diet, a low-intensity resistance exercise training (LIRET) program, or a combination of both for 12 weeks, and they had their PWV (aortic, brachial–ankle and femoral–ankle) measured, among other parameters. Regarding aortic PWV, the within-group analysis showed no difference (LIRET: *p* = 0.55; diet: *p* = 0.29; diet+LIRET: *p* = 0.26). The same was applied to the between-group analysis for aortic PWV (*p* = 0.99). However, baPWV was found to be reduced with both diet (*p* = 0.04) and diet + LIRET intervention (*p* = 0.01), whereas faPWV decreased only with diet (*p* = 0.01) [68].

Volek and colleagues [69] performed a randomized, controlled dietary intervention trial lasting for 12 weeks which included adults with overweight and atherogenic dyslipidemia. They aimed to compare a carbohydrate-restricted diet (CRD) to a low-fat diet (LFD) (both hypocaloric), regarding their effect on FMD. Study results indicated that after 12 weeks of intervention, peak FMD at 3 h elevated from 5.1% ± 2.7% to 6.5% ± 3.3% in the CRD group and decreased from 7.9% ± 5.3% to 5.2% ± 2.9% in the LFD group (*p* = 0.004 for diet × time interaction) [69].

A longitudinal, randomized, open study design was developed by Buscemi and colleagues [70] including subjects with overweight or obesity. Participants were assigned to receive either an Atkins-type low carbohydrate diet (ALCD) or a hypocaloric MD (HMD). For the assessment of EF, baFMD was measured three times; the first time was before the enrollment (T0), the second time was 5−7 days after the diet was started (T5), and the third time was after 2 months (T60). FMD was found to be lower in the HMD group when compared to the ALCD group in T5 (14.5 ± 2.8 vs. 5.2 ± 0.8; *p* = 0.005). Additionally, a difference was observed in the FMD between the two groups among the T0 and the T5 (Δ T0-T5) timepoint (4.2 ± 1.5 vs. −7.0 ± 2.5; *p* = 0.001) [70].

The characteristics of the included studies assessing the effect of the CR approaches on vascular health markers are presented in Table 4.

### 3.5. Low-Carbohydrate Diet

Previous studies have reported that carbohydrate consumption, particularly high sucrose diets, may have a detrimental effect on arterial stiffness through mechanisms associated with hyperglycemia [72]. Furthermore, the data indicate that impaired postprandial blood glucose, as opposed to fasting blood glucose, exerts a more pronounced impact on CVD development [73].

Gram-Kampmann et al. [74] conducted an outpatient, open-label RCT including patients with T2DM. Participants received either a low-carbohydrate diet (LCaD) (20% of energy consisting of carbohydrates) or a control diet (50−60% of energy consisting of carbohydrates) and had their FMD and nitroglycerine-induced vasodilation (NID) evaluated as markers of EF. No effects were observed among the studied parameters (FMD: *p* = 0.34; NID: *p* = 0.53).

In the open-label nRCT by Athinarayanan et al. [75], patients with T2DM followed a very LCaD or received UC and had their cIMT measured. In the intervention group, cIMT remained unaffected during the 2-year timeframe (95% CI: −0.07 to 0.01).

Hwang and colleagues [76], in their prospective randomized parallel design clinical trial, enrolled healthy women with obesity who were asked to follow a 6-week LCaD (10% of energy by carbohydrates) with or without CR. Macrovascular EF was assessed by baFMD and NID. Following the 6-week period, no associations were observed regarding FMD (p_Group×Time_ = 0.4) and NID (p_Group×Time_ = 0.9).

The CT by Syed-Abdul et al. [77] included participants with characteristics of insulin resistance (IR) and MetS who followed a LCaD for 4 weeks and had their PWV measured as a parameter of AS. After the intervention, PWV was found to be decreased in the whole group (*p* = 0.008) as well as in women (*p* = 0.028), but not in men (*p* = 0.144).

McDonald and colleagues [78] evaluated the cIMT in adult participants with epilepsy following a modified Atkins diet (MAD) for >1 year, compared with patients with epilepsy who have never tried diet therapy, in an nRCT design. No difference was observed in cIMT between groups (left cIMT: *p* = 0.695; right cIMT: *p* = 0.473; cIMt > 75th percentile left: *p* = 0.741; cIMT > 75th percentile right: *p* = 0.191).

In the study by Keogh et al. [79], participants with abdominal obesity received either a caloric-restricted very low-carbohydrate, high-saturated-fat diet (VLCHSFD) or an isocaloric conventional high-carbohydrate, low-saturated-fat diet (HCLSFD) for a time interval of 8 weeks. AIx, FMD, and PWV were assessed as markers of vascular function. FMD, PWV, and AIx remained unchanged with both dietary patterns.

The characteristics of the included studies assessing the effect of the low-carbohydrate diet on vascular health markers are presented in Table 5.

### 3.6. Low-Fat Diet

Low-fat diets have been associated with a positive impact on vascular health, as this dietary regimen seems to reduce the risk factors related to cardiovascular disease. Excessive fat mass has also been associated with abnormal EF characterized by reduced vasodilation and an increased blood flow [65].

The randomized crossover study design by Fryer and colleagues [80] consisted of healthy male participants who consumed either a high-fat (HFM) or a low-fat meal (LFM) during each study visit. CfPWV and faPWV were evaluated as parameters of AS. No associations were found for faPWV, whereas an increase in cfPWV was observed after the consumption of the HFM (post hoc MD = 0.59; 95% CI: 0.29 to 0.89 m/s).

Mohler 3rd et al. [81] conducted a multicenter RCT including subjects with obesity who were assigned to either an LCaD or an LFD and had their FMD measured, as an index of EF. The findings showed no differences between the two diets in FMD at any time point (week 0, *p* = 0.17; 3 months, *p* = 0.66; 6 months, *p* = 0.80; 1 year, *p* = 0.86; 2 years, *p* = 0.29).

In the RCT by Davis and colleagues [82], patients with T2DM were randomized to either receive a LCaD or an LFD. soluble intercellular adhesion molecule (sICAM) and soluble E-selectin were measured in order to evaluate the EF. In the LCaD group, a reduction in sICAM (±S.E.) from 234 ± 22 to 199 ± 23 (*p* = 0.001) and in soluble E-selectin from 93 ± 10 to 82 ± 10 (*p* = 0.05) were observed, while no changes were observed in the LFD group.

Varady and colleagues [83] performed a 6-week randomized, parallel-arm, dietary intervention trial including participants with obesity who received either an HFD or an LFD and had their baFMD evaluated. The LFD was found to be associated with an increase in baFMD (*p* < 0.05), while the HFD was found to be associated with a reduction in the aforementioned parameter (*p* < 0.05).

In the study by Lambert et al. [84], healthy young individuals comprised the population of interest, and were classified into tertiles based on their habitual saturated fat (SF) consumption. The studied endpoint was EF evaluated by digital amplitude tonometry using the reactive hyperemia index (RHI). The study showed that participants classified in the high tertile of saturated fat intake presented impaired EF compared to the other tertiles (High SF vs. medium SF: 1.60 ± 0.08 vs. 2.23 ± 0.16; high SF vs. low SF: 1.60 ± 0.08 vs. 2.12 ± 0.14, *p* < 0.01).

The characteristics of the included studies assessing the effect of the low-fat diet on vascular health markers are presented in Table 6.

### 3.7. Western Diet

The Western diet is known for its high content of saturated and trans fatty acids, which are closely related to the development of atherosclerosis and the impairment of the function and structure of blood vessels. This dietary pattern is also characterized by a high consumption of processed foods and it has been associated with obesity and insulin resistance. Additionally, beneficial dietary components that reduce oxidative stress and inflammation are not present in this diet. Several studies have investigated the association of AS and endothelial function in mice, whereas studies in humans are limited [85,86].

In the RoCAV study described before, the Western-type dietary pattern (PC1) was correlated to elevated cfPWV in the first (+0.29 m/s for 1 SD increase of PC1, 95% CI: 0.08 to 0.50) and the second model (+0.29 m/s, 95% CI: 0.08 to 0.50). The association remained also in the third and fourth model (+0.31 m/s, 95% CI: 0.11 to 0.52; +0.24 m/s, 95% CI: 0.03 to 0.45, respectively) [33] (Table 7).

## 4. Discussion

The objective of the present narrative review was to synthesize the available literature concerning the effect of different dietary patterns on vascular health. According to the published literature, the MD, DASH, and CR diets may have a beneficial effect on vascular health.

Regarding the MD, the published SR&MA reported a favorable impact on EF and FMD. Furthermore, the majority of the available RCTs indicated that the MD was superior to the control groups, including habitual diet or LFD, in improving endpoints such as cIMT, FMD, and PWV. In contrast, conflicting findings were observed in the published CT concerning outcomes related to vascular health.

Studies examining the effect of the DASH diet on indicators of vascular health and endothelial function yielded mixed results, with half supporting a beneficial effect, while the remaining half reporting no difference compared to the control group.

As far as the VegD is concerned, the majority of the available literature suggests inefficacy in ameliorating vascular health and endothelial function markers.

Conversely, a CR diet appears to confer benefits related to arterial stiffness and endothelial function, supported by the published SR&MA, RCTs, and CTs on this topic.

Data pertaining to both LFD and LCD indicated their ineffectiveness for the improvement of the studied endpoints, particularly the cIMT, FMD, and PWV.

Last but not least, the Western-type diet appears to have a detrimental effect on arterial stiffness, as assessed by the gold-standard method, namely cfPWV.

It is noteworthy to acknowledge the considerable heterogeneity among the published studies within each dietary pattern, rendering the derivation of definite conclusions challenging. Diversities exist not only in the studied populations but also in the nature of interventions employed across studies. Moreover, the majority of the included studies exhibit short durations, coupled with a lack of post-intervention follow-up visits to assess arterial stiffness and endothelial function markers. Lastly, long-term nutritional RCTs encounter challenges due to participants’ poor adherence to the prescribed diets [87], prompting the adoption of alternative study designs such as observational studies. This shift introduced potential limitations to the findings’ credibility. A body of evidence indicates that dietary adherence is a critical factor in enhancing the effectiveness of dietary interventions [88] and, hence, designates the potential role of nutrition as a therapeutic strategy for disease management. However, a significant proportion of dietary trials neglect to report participants’ adherence to the intervention.

## 5. Conclusions

Based on the existing evidence, the MD, DASH, and calorie restriction diets may have a beneficial effect on vascular health. More randomized controlled trials with sufficient sample sizes, longer follow-ups, rigorous methodologies, and, possibly, head-to-head comparisons between the different diets are welcomed to shed light on this topic.

## Figures and Tables

**Table 1:** Characteristics of studies including the Mediterranean diet.

Reference	Study Design	Population	Interventions	Outcomes	Findings *
Shannon et al., 2020 [38]	SR and MA of RCTs	14 RCTs (*n* = 1930)	Arm 1: MD Arm 2: Control	EF and FMD	MD ⭢ ⭡ EF andFMD
Rallidis et al., 2009 [25]	RCT	P with AO without CVD or T2DM (*n* = 99)	Arm 1: MD supervised by a dietitian Arm 2: MD	FMD	MD with supervision ⭢ ⭡ FMD
Murie-Fernandez et al., 2011 [37]	RCT	High CVD risk adults (*n* = 187)	Arm 1: MD + EVOO Arm 2: MD + Nuts Arm 3: Control diet	cIMT	Arms 1 and 2 (alone or merged) ⭢ ⭣ cIMT when cIMT ≥ 0.9 mm and ⭤ cIMT when cIMT < 0.9 mm
Klonizakis et al., 2013 [19]	RCT	Healthy adults (*n* = 22)	Arm 1: MD + Exercise Arm 2: Exercise	SNP	MD ⭢ ⭤ SNP compared to exercise alone
Lee at al., 2015 [35]	RCT CO	Healthy women (*n* = 24)	Arm 1: MD Arm 2: Habitual diet	AIx	MD ⭢ ⭤ AIx
Davis et al., 2017 [36]	RCT	Healthy older adults (*n* = 152)	Arm1: MD Arm 2: Habitual diet	FMD	MD ⭢ ⭡ FMD
Maiorino et al., 2017 [30]	RCT	Newly diagnosed T2DM (*n* = 215)	Arm 1: MD Arm 2: LFD	cIMT	MD ⭢ ⭣ cIMT compared to LFD
Torres-Peña et al., 2018 [27]	RCT	P with CHD (*n* = 805)	Arm 1: MD Arm 2: LFD	FMD	MD ⭢ ⭡ FMD in patients with CHD and T2DM compared to LFD
Jennings et al., 2019 [9]	RCT	Older adults (*n* = 1250)	Arm 1: Personalized MD Arm 2: Habitual diet	AIx and PWV	MD ⭢ ⭡ AIx MD ⭢ ⭤ PWV
Yubero- Serrano et al., 2020 [26]	RCT	P with CHD (*n* = 805)	Arm 1: MD Arm 2: LFD	FMD	MD ⭢ ⭡ FMD compared to LFD LFD ⭢ ⭤ FMD
Lydakis et al., 2012 [39]	CS	Healthy children (*n* = 277)	Adherence to MD (KIDMED)	AIx	1 point ⭡ in the KIDMED ⭢ ⭣ AIx
Rodríguez-Martin et al., 2017 [24]	CS	P without CVD (*n* = 1553)	Adherence (EVIDENT diet index)	PWV	1 point ⭡ in the EVIDENT index ⭢ ⭣ PWV
García-Hermoso et al., 2018 [23]	CS	Adults (*n* = 1365)	Adherence (MEDAS test)	AASIx, cAIx75, PWV, rAIx75	⭡ in the MEDAS test ⭢ ⭤ CAIx75 and AASIx ⭡ in the MEDAS test ⭢ ⭣ PWV and rAIx75
Sánchez et al., 2020 [29]	CS	P without CVD (*n* = 501)	Adherence (MEDAS test)	VAI (incidence of EVA)	⭡ in the MEDAS test ⭢ ⭣ incidence of EVA
Angelis et al., 2021 [34]	CS	Males with CHF (*n* = 150)	Adherence (MedDietScore)	AIx, cIMT, PWV	⭡ in the MedDietScore ⭢ ⭣ AIx and cIMT ⭡ in the MedDietScore ⭢ ⭤ PWV
Lasalvia et al., 2021 [33]	CS	Healthy adults (*n* = 3777)	Adherence (PCA and MedS)	cfPWV	Adherence to the MD ⭢ ⭤ cfPWV in any model
Gómez-Sánchez et al., 2022 [28]	CS	P with moderate CVD risk (*n* = 2475)	Adherence to MD	baPWV and VAI	1 point ⭡ in the MD adherence ⭢ ⭣ baPWV ⭡ in the MD adherence ⭢ ⭣ incidence of EVA
Lobene et al., 2022 [21]	CS	Healthy young adults (*n* = 56)	Adherence (aMED Score)	AIx, FMD, PWV	1 point ⭡ in the MD adherence ⭢ ⭣ AIx 1 point ⭡ in the MD adherence ⭢ ⭤ FMD and PWV

⭡: increase; ⭣: decrease; ⭤: no difference. * In the RCTs, the findings are reported compared to the control group, unless otherwise stated. AASIx: ambulatory arterial stiffness index; AIx: augmentation index; aMED: alternative Mediterranean diet; AO: abdominal obesity; baPWV: brachial–ankle pulse wave velocity; cAIx75: central augmentation index75; cIMT: carotid intima-media thickness; cfPWV: carotid–femoral pulse wave velocity; CHD: coronary heart disease; CHF: chronic heart failure; CO: crossover; CS: cross-sectional; CVD: cardiovascular disease; EF: endothelial function; EVA: early vascular aging; EVOO: extra-virgin olive oil; FMD: flow-mediated dilatation; KIDMED: Mediterranean Diet Quality Index for Children and Adolescents; LFD: low-fat diet; MA: meta-analysis; MD: Mediterranean diet; MEDAS: Mediterranean diet adherence screener; MedS: Mediterranean diet adherence score; P: participants; PCA: principal component analysis; PWV: pulse wave velocity; rAIx75: Radial Augmentation Index75; RCT: randomized controlled trial; SNP: sodium nitroprusside; SR: systematic review; T2DM: type 2 diabetes mellitus; VAI: vascular arterial index.

**Table 2:** Characteristics of studies including the DASH diet.

Reference	Study Design	Population	Interventions	Outcomes	Findings *
Blumenthal et al., 2010 [45]	RCT (ENCORE Study)	Overweight or obese unmedicated outpatients with high BP (*n* = 144)	Arm 1: DASH-A Arm 2: DASH-WM Arm 3: Habitual diet	FMD and PWV	DASH-A and DASH-WM ⭢ ⭣ PWV DASH-WM ⭢ ⭣ PWV compared to DASH-A DASH-A and DASH-WM ⭢ ⭤ FMD
Lin et al., 2012 [49]	RCT	P with unmedicated stage 1 HTN (*n* = 20)	Arm 1: DASH Diet Arm 2: Control diet	AIx, baFMD, PWV	DASH ⭢ ⭤ AIx and baFMD DASH ⭢ ⭣ PWV over time
Blumenthal et al., 2021 [48]	RCT (TRIUMPH Study)	P with resistant HTN (*n* = 1040)	Arm 1: C-LIFE + DASH diet Arm 2: SEPA + DASH diet	FMD and PWV	C-LIFE ⭢ ⭤ FMD and PWV SEPA ⭢ ⭣ FMD
Gauci et al., 2022 [46]	CS	Middle-aged adults (*n* = 141)	Adherence (Folsom DASH score)	AIx and PWV	1 point ⭡ in the DASH diet adherence ⭢ ⭣ AIx Adherence to the DASH diet ⭢ ⭤ PWV
Lobene et al., 2022 [21]	CS	Healthy young adults (*n* = 56)	Adherence (Fung DASH score and Mellen DASH score)	AIx, FMD, PWV	Adherence to the DASH diet ⭢ ⭤ AIx, FMD, PWV
Maddock et al., 2018 [47]	Cohort	Participants (*n* = 1409)	Adherence (Fung DASH score)	cIMT and PWV	⭡ in the DASH diet adherence ⭢ ⭣ scIMT ⭡ in the DASH diet adherence ⭢ ⭣ sPWV

⭡: increase; ⭣: decrease; ⭤: no difference. * In the RCTs, the findings are reported compared to the control group, unless otherwise stated. AIx: augmentation index; baFMD: brachial artery flow-mediated dilatation; baPWV: brachial–ankle pulse wave velocity; BP: blood pressure; cIMT: carotid intima-media thickness; C-LIFE: Center-based Lifestyle Intervention; CS: cross-sectional; DASH: Dietary Approaches to Stop Hypertension; DASH-A: DASH diet alone; DASH-WM: DASH diet with behavioral weight management program; FMD: flow-mediated dilatation; HTN: hypertension; P: participants; PWV: pulse wave velocity; RCT: randomized controlled trial; scIMT: standardized cIMT; SEPA: Standardized Education and Physician Advice; sPWV: standardized PWV.

**Table 3:** Characteristics of studies including the vegetarian diet.

Reference	Study Design	Population	Interventions	Outcomes	Findings *
Gonzalez et al., 2022 [50]	CS	Normotensive young healthy adults (*n* = 58)	Arm 1: VegD Arm 2: OmnD	AIx, baFMD, PWV	⭤ AIx, baFMD and PWV between the two groups
Mayra et al., 2022 [51]	CS	Healthy non-smoking adults (*n* = 55)	Arm 1: VegD/VgD Arm 2: Omn-D	cfPWV	⭤ cfPWV between the two groups
Page et al., 2022 [53]	CS	Young healthy men (*n* = 25)	Arm 1: VgD Arm 2: OmnD	baFMD and cIMT	⭤ baFMD and cIMT between the two dietary patterns
Chen et al., 2019 [52]	Prospective	Participants (52% vegetarians) (*n* = 985)	-	cIMT	Vegetarians ⭢ ⭣ cIMT compared to the rest

⭣: decrease; ⭤: no difference. * In the RCTs, the findings are reported compared to the control group, unless otherwise stated. AIx: augmentation index; baFMD: brachial artery flow-mediated dilatation; cIMT: carotid intima-media thickness; cfPWV: carotid–femoral pulse wave velocity; CS: cross-sectional; OmnD: omnivore diet; PWV: pulse wave velocity; VegD: vegetarian diet; VgD: vegan diet.

**Table 4:** Characteristics of studies including the calorie restriction diet.

Reference	Study Design	Population	Interventions	Outcomes	Findings *
Petersen et al., 2015 [63]	SR and MA	20 studies, *n* = 1259	Diet for weight loss ± Exercise ± Drugs	PWV, baPWV, cfPWV	Diet for weight loss ± Exercise ± Drugs ⭢ ⭣ PWV
Buscemi et al., 2009 [70]	RCT	Women with OW or OB (*n* = 28)	Arm 1: ALCD Arm 2: MHD	baFMD	MHD ⭢ ⭡ FMD compared to ALCD at T5 MHD ⭤ FMD compared to ALCD at T60
Volek et al., 2009 [69]	RCT	Adults with OW and AD (*n* = 40)	Arm 1: CRD Arm 2: LFD	FMD	CRD ⭢ ⭡ FMD ⭣ FMD ⭢ in the LFD group
Figueroa et al., 2013 [68]	RCT	PM women with OW or OB (*n* = 41)	Arm 1: HD Arm 2: LIRET Arm 3: HD + LIRET	baPWV and PWV	HD ⭢ ⭣ baPWV HD + LIRET ⭢ ⭣ baPWV LIRET ⭢ ⭤ baPWV
Klempel et al., 2013 [64]	RCT	P with obesity (*n* = 32)	Arm 1: ADF-HF (45% fat) Arm 2: ADF-LF diet (25% fat)	baFMD	ADF-LF ⭢ ⭡ FMD compared to ADF-HF
Gonçalinho et al., 2021 [71]	RCT	Healthy adults (*n* = 48)	Arm 1: Resveratrol supplement Arm 2: LCD	FMD and NMD	⭤ FMD and NMD between groups
Jefferson, 2016 [60]	RCT	Older adults with OW or OB (*n* = 32)	Arm 1: SMIRT Arm 2: RTCR	baPWV	⭤ baPWV within groups
Weiss et al., 2016 [66]	RCT	OW and sedentary adults (*n* = 52)	Arm 1: CR program Arm 2: EX program Arm 3: CREX	AIx and PWV	⭤ AIx and PWV between and within groups
Headland et al., 2018 [59]	RCT CO	Healthy adults (*n* = 35)	2 days VLED/ 5 days habitual eating	FMD	⭤ FMD
Nordstrand et al., 2013 [67]	nRCT	P with MO (*n* = 179)	Arm 1: LCD Arm 2: ILI	PWV	ILI ⭢ ⭣ PWV compared to LCD LCD ⭢ ⭤ PWV
Raitakari et al., 2004 [61]	CT	Adults with OW (*n* = 67)	LCD	FMD and NMD	LCD ⭢ ⭡ FMD and NMD
Alinezhad-Namaghi et al., 2023 [58]	Cohort	Adults with MetS (*n* = 95)	Arm 1: RF Arm 2: RNF	Arterial age, cAlx (%), cAIx, cAP, PVW	RF ⭢ ⭣ arterial age and cAP compared to RNF RF ⭢ ⭤ cAIx (%), cAIx, PWV compared to RNF

⭡: increase; ⭣: decrease; ⭤: no difference. * In the RCTs, the findings are reported compared to the control group, unless otherwise stated. ADF-HF: alternate day fasting high fat; ADF-LF: alternate day fasting low fat; AIx: augmentation index; ALCD: Atkins low carbohydrate diet; baFMD: brachial artery flow-mediated dilatation; baPWV: brachial–ankle pulse wave velocity; cAIx: central augmentation index; cAP: central augmentation pressure; cfPWV: carotid–femoral pulse wave velocity; CO: crossover; CR: caloric restriction; CRD: carbohydrate-restricted diet; CREX: calorie restriction combined with endurance exercise; CS: cross-sectional; CT: clinical trial; faPWV: femoral–ankle pulse wave velocity; FMD: flow-mediated dilatation; HD: hypocaloric diet; ILI: intensive lifestyle intervention program; LCD: low calorie diet; LFD: low-fat diet; LIRET: low-intensity resistance exercise training program; MA: meta-analysis; MetS: metabolic syndrome; MHD: Mediterranean hypocaloric diet; MO: morbid obesity; NMD: nitrate-mediated dilation; nRCT: non-randomized controlled trial; OB: obesity; OmnD: omnivore diet; OW: overweight; P: participants; PM: post-menopausal; PWV: pulse wave velocity; RCT: randomized controlled trial; RF: Ramadan fasting; RNF: Ramadan non-fasting; RTCR: resistance training caloric restriction; SMIRT: supervised moderate-intensity resistance training; SR: systematic review; T5: 5−7 days; T60: 2 months; VLED: very low-energy diet.

**Table 5:** Characteristics of studies including the low-carbohydrate diet.

Reference	Study Design	Population	Interventions	Outcomes	Findings *
Keogh et al., 2008 ** [79]	RCT	P with AO (*n* = 99)	Arm 1:VLCHSFD Arm 2: HCLSFD	AIx, baFMD, PWV	⭤ AIx, baFMD within groups ⭣ PWV within groups
Hwang et al., 2020 [76]	RCT	Healthy women with obesity (*n* = 21)	Arm 1: LCaD + CR Arm 2: LCaD w/o CR	baFMD and NID	⭤ baFMD and NID
Gram-Kampmann et al., 2023 [74]	RCT	P with T2DM (*n* = 71)	Arm 1: LCaD Arm 2: CD	FMD and NID	⭤ FMD and NID between and within groups
Athinarayanan et al., 2020 [75]	nRCT	P with T2DM (*n* = 262)	Arm 1: LCaD Arm 2: UC	cIMT	⭤ cIMT
McDonald et al., 2018 [78]	nRCT	P with epilepsy (*n* = 41)	Arm 1: MAD for >1 year Arm 2: Naïve to MAD	cIMT	⭤ cIMT between groups
Syed-Abdul et al., 2018 ** [77]	CT	P with characteristics of IR and MetS (*n* = 20)	LCaD	cfPWV	LCaD ⭢ ⭣ PWV in women not men

⭣: decrease; ⭤: no difference. * In the RCTs, the findings are reported compared to the control group, unless otherwise stated. ** There was also a CR intervention. AIx: augmentation index; AO: abdominal obesity; baFMD: brachial artery flow-mediated dilatation; CD: control diet; cfPWV: carotid–femoral pulse wave velocity; cIMT: carotid intima-media thickness; CR: calorie restriction; CT: clinical trial; FMD: flow-mediated dilatation; HCLSFD: high carbohydrate, low-saturated-fat diet; IR: insulin resistance; LCaD: low-carbohydrate diet; MAD: modified Atkins diet; MetS: metabolic syndrome; NID: nitroglycerine-induced vasodilation; nRCT: non randomized controlled trial; P: participants; RWV: pulse wave velocity; RCT: randomized controlled trial; T2DM: type 2 diabetes mellitus; UC: usual care; VLCHSFD: very-low-carbohydrate high-saturated-fat diet; W/O: without.

**Table 6:** Characteristics of studies including the low-fat diet.

Reference	Study Design	Population	Interventions	Outcomes	Findings *
Davis et al., 2011 ** [82]	RCT	P with T2DM (*n* = 27)	Arm 1: LCaD Arm 2: LFD	E-selectin and sICAM	LCaD ⭢ ⭣ E-selectin and sICAM LFD ⭢ ⭤ E-selectin and sICAM
Varady et al., 2011 ** [83]	RCT	P with OB (*n* = 17)	Arm 1: LFD Arm 2: HFD	baFMD	LFD ⭢ ⭡ baFMD HFD ⭢ ⭣ baFMD
Mohler 3rd et al., 2013 ** [81]	RCT	P with OB (*n* = 121)	Arm 1: LCaD Arm 2: LFD	FMD	⭤ FMD between and within groups
Fryer et al., 2021 [80]	RCT CO	Healthy males (*n* = 13)	Arm 1: HFM Arm 2: LFM	cfPWV and faPWV	HFM ⭢ ⭡ cfPWV compared to LFM ⭤ faPWV
Lambert et al., 2017 [84]	Observational	P with OW	Tertiles of saturated fat/total fat	RHI	High tertile ⭢ ⭣ RHI compared to lower tertiles

⭡: increase; ⭣: decrease; ⭤: no difference. * In the RCTs, the findings are reported compared to the control group, unless otherwise stated. ** There was also a CR intervention. AIx: augmentation index; baFMD: brachial artery flow-mediated dilatation; CO: crossover; CR: calorie restriction; faPWV: femoral–ankle pulse wave velocity; FMD: flow-mediated dilatation; HFD: high-fat diet; HFM: high-fat meal; LCaD: low-carbohydrate diet; LFD: low-fat diet; LFM: low-fat meal; OB: obesity; OW: overweight; P: participants; PWV: pulse wave velocity; RCT: randomized controlled trial; RHI: reactive hyperemia index; SFA: saturated fatty acid; sICAM: soluble intercellular adhesion molecule; T2DM: type 2 diabetes mellitus.

**Table 7:** Characteristics of studies including the Western diet.

Reference	Study Design	Population	Interventions	Outcomes	Findings
Lasalvia et al., 2021 [33]	CS	P with chronic diseases (*n* = 3777)	Arm 1: WTD (PC1) Arm 2: MD (PC2)	cfPWV	WTD ⭢ ⭡ cfPWV in all models

⭡: increase; cfPWV: carotid–femoral pulse wave velocity; CS: cross-sectional; MD: Mediterranean diet; P: participants; PC: principal component; WTD: Western-type diet.

## Data Availability

Data are contained within the article and Supplementary Materials.

## Supplementary Materials

The following supporting information can be downloaded at https://www.mdpi.com/article/10.3390/life14020267/s1, Table S1: Search strategy for identifying studies on PubMed.

## Abbreviations

AASIx	Ambulatory arterial stiffness index
ADF	Alternate day fasting
ADF-HF	Alternate day fasting high fat
ADF-LF	Alternate day fasting low fat
AIx	Augmentation index
ALCD	Atkins low-carbohydrate diet
aMED	Alternative Mediterranean diet
AMPK	AMP-activated protein kinase
AO	Abdominal obesity
baFMD	Brachial artery flow-mediated dilatation
baPWV	Brachial–ankle pulse wave velocity
BMI	Body mass index
BP	Blood pressure
cAIx	Central augmentation index
cAIx75	Central Augmentation Index75
CD	Control diet
cfPWV	Carotid–femoral pulse wave velocity
CHD	Coronary heart disease
CHF	Chronic heart failure
cIMT	Carotid intima-media thickness
C-LIFE	Center-based lifestyle intervention
CO	Crossover
CR	Caloric restriction
CRD	Carbohydrate-restricted diet
CREX	Calorie restriction combined with endurance exercise
CS	Cross-sectional
CT	Clinical trial
CVD	Cardiovascular disease
DASH-A	DASH diet alone
DASH-WM	DASH diet with behavioral weight management program
EF	Endothelial function
EVA	Early vascular aging
EVOO	Extra-virgin olive oil
ΕΧ	Exercise program
faPWV	Femoral–ankle pulse wave velocity
FMD	Flow-mediated dilatation
HD	Hypocaloric diet
HFD	High-fat diet
HFM	High-fat meal
HMD	Hypocaloric
HTN	Hypertension
ILI	Intensive lifestyle intervention program
IR	Insulin resistance
KIDMED	Mediterranean Diet Quality Index for Children and Adolescents
LCaD	Low-carbohydrate diet
LCD	Low-calorie diet
LFD	Low-fat diet
LFM	Low-fat meal
LIRET	Low-intensity resistance exercise training program
MA	Meta-analysis
MAD	Modified Atkins diet
MAST	Memory and attention supplement trial
MD	Mediterranean diet
MEDAS	Mediterranean diet adherence screener
MedS	Mediterranean diet adherence score
MetS	Metabolic syndrome
MHD	Mediterranean hypocaloric diet
MIND	Mediterranean-DASH Intervention for Neurodegenerative Delay
MO	Morbid obesity
MRC	Medical Research Council
mTOR	Mammalian target of rapamycin
MUFA	Monounsaturated fatty acid
NHSD	National Survey of Health and Development
NID	Nitroglycerine-induced vasodilation
NMD	Nitrate-mediated dilatation
nRCT	Non-randomized controlled trial
OB	Obesity
OmnD	Omnivore diet
OW	Overweight
P	Participants
PC	Principal component
PCA	Principal component analysis
PM	Post-menopausal
PWV	Pulse wave velocity
RAAS	Renin–angiotensin–aldosterone system
rAIx75	Radial Augmentation Index75
RCT	Randomized controlled trial
RF	Ramadan fasting
RHI	Reactive hyperemia index
RNF	Ramadan non-fasting
RTCR	Resistance training caloric restriction
SEPA	Standardized Education and Physician Advice
SFA	Saturated fatty acid
sICAM	Soluble intercellular adhesion molecule
SIRT−1	Sirtuin−1
SMD	Standardized mean difference
SMIRT	Supervised moderate-intensity resistance training
SNP	Sodium nitroprusside
SR	Systematic review
T2DM	Type 2 diabetes mellitus
UC	Usual care
VAI	Vascular arterial index
VegD	Vegetarian diet
VgD	Vegan diet
VLCD	Very-low-caloric diet
VLED	Very-low-energy diet
WTD	Western-type diet
