# Supplementary material for: The Effect of Diet on Vascular Aging: A Narrative Review of the Available Literature
Source: Life (Basel). 2024 Feb 17;14(2):267. doi: 10.3390/life14020267 (PMC10890697; doi:10.3390/life14020267)
Supplement: Supplementary file 1 [file life-14-00267-s001.zip › life-2804321-supplementary.pdf]

Contents

Supplementary Table S1. Search strategy for identifying studies on PubMed

Supplementary Table S1. Search strategy for identifying studies on PubMed

Search	Query	Results
#1	Mediterranean diet [MeSH]	5446
#2	Mediterranean diet [tw]	7879
#3	DASH diet [MeSH]	421
#4	DASH diet [tw]	846
#5	Dietary approaches to stop hypertension [tw]	1355
#6	Vegetarian diet [MeSH]	4175
#7	Vegetarian diet [tw]	1482
#8	Vegan diet [tw]	647
#9	Western diet [MeSH]	1493
#10	Western diet [tw]	4189
#11	Low fat diet [MeSH]	4046
#12	Low fat diet [tw]	4549
#13	Low-fat diet [tw]	4549
#14	Low fat [tw]	13244
#15	Fat restricted [tw]	4151
#16	Fat restricted diet* [tw]	106
#17	Fat-restricted diet* [tw]	106
#18	Reduced fat [tw]	1907
#19	Low carbohydrate diet [MeSH]	4425
#20	Low carbohydrate diet [tw]	1679
#21	Low CHO diet [tw]	79
#22	Carbohydrate restricted [tw]	2341
#23	Carbohydrate restricted diet* [tw]	210
#24	Carbohydrate-restricted diet* [tw]	210
#25	Reduced carbohydrate [tw]	285
#26	Energy restriction diet [MeSH]	8626

#27	Energy restriction diet* [tw]	36
#28	Energy restricted [tw]	1030
#29	Calorie restriction diet [MeSH]	4716
#30	Calorie restriction diet* [tw]	53
#31	Calori* restricted [tw]	4102
#32	Weight loss [tw]	124254
#33	#1-#32/OR	170044
#34	Vascular health [tw]	2063
#35	Arterial stiffness [MeSH]	8388
#36	Arterial stiffness [tw]	12302
#37	Endothelial function [MeSH]	686
#38	Endothelial function [tw]	21357
#39	Pulse wave velocity [MeSH]	6683
#40	Pulse wave velocity [tw]	11826
#41	Flow-mediated dilatation [MeSH]	168
#42	Flow-mediated dilatation [tw]	2149
#43	Augmentation index [MeSH]	15
#44	Augmentation index [tw]	3545
#45	Vascular aging [MeSH]	16706
#46	Vascular aging [tw]	1268
#47	Carotid intima media thickness [MeSH]	6903
#48	Carotid intima media thickness [tw]	11010
#49	Carotid intima-media thickness [tw]	11010
#50	cIMT [tw]	4744
#51	#34-#49/OR	68045
#52	#33 AND #51	1123
The last search was performed on December 27th 2023		
